# Evaluating the Effectiveness of Ibuprofen Versus Acetaminophen in Closing Patent Ductus Arteriosus in Preterm Neonates

**DOI:** 10.3390/children13020257

**Published:** 2026-02-12

**Authors:** Shaimaa Alsulami, Mona Aljehani, Najla Alotaibi, Mohammed Y. Al-Hindi, Mohammed Alharbi

**Affiliations:** 1 Pharmaceutical Practices Department, Faculty of Pharmacy, Umm Al-Qura University, Makkah 24382, Saudi Arabia; 2 Department of Pharmaceutical Care Services, King Abdulaziz Medical City, Jeddah 21423, Saudi Arabia; 3 King Abdullah International Medical Research Center (KAIMRC), Jeddah 21423, Saudi Arabia; 4 Population Health Management, Jeddah Second Health Cluster, Ministry of Health, Jeddah 23816, Saudi Arabia; 5 Neonatal Intensive Care Department, King Abdullah Specialized Children’s Hospital, Jeddah 22384, Saudi Arabia; 6 College of Medicine, King Saud bin Abdulaziz University for Health Sciences, Jeddah 21423, Saudi Arabia

**Keywords:** patent ductus arteriosus, neonates, ibuprofen, acetaminophen

## Abstract

**Background/Objective:** Patent Ductus Arteriosus (PDA) is a common congenital heart defect causing high morbidity and mortality in preterm neonates. IV ibuprofen is the standard treatment, with acetaminophen as a potential alternative when ibuprofen is contraindicated. However, evidence for acetaminophen’s effectiveness is inconclusive. This study aimed to compare the efficacy and safety of IV acetaminophen versus IV ibuprofen as the initial treatment for PDA closure in preterm neonates. **Methods:** A retrospective cohort study was conducted at a tertiary Saudi hospital. This study included preterm neonates with a gestational age of ≤32 weeks diagnosed with PDA and treated with IV ibuprofen or IV acetaminophen. The primary outcome was to evaluate the efficacy of ibuprofen versus acetaminophen for treating PDA. **Results:** A total of 95 courses were included. Of these, 49 neonates received ibuprofen, and 18 neonates received acetaminophen as first therapy. The mean age at the initial course was 5.47 ± 10.30 days for the ibuprofen group and 5.22 ± 6.43 days for the acetaminophen group. In most neonates, the hemodynamic significance of the PDA was confirmed by ultrasound examination. As a result, 35 of 49 neonates treated with ibuprofen experienced successful full PDA closure, with a rate of 71.4%, compared to 10 of 18 in the acetaminophen group, which had a rate of 55.6%. However, this difference was not statistically significant (*p*-value = 0.35). **Conclusions:** A trend toward higher PDA closure with ibuprofen was observed compared to acetaminophen, without a statistically significant difference. Both treatments showed comparable safety. Further studies are needed to confirm these findings and optimize acetaminophen dosing.

## 1. Introduction

Patent Ductus Arteriosus (PDA) represents the most common congenital cardiovascular anomaly among preterm neonates. PDA is characterized by failure of the ductus arteriosus to close following birth. This failure may lead to the persistence of a direct connection between the proximal descending aorta and the main pulmonary artery, causing significant hemodynamic instability in preterm neonates. The incidence of PDA in extremely preterm neonates (26 weeks’ gestational age) ranges from 30 to 67% [1,2].

Untreated PDA is associated with serious clinical issues; decreased pulmonary vascular resistance (PVR) results in increased left-to-right shunting through the ductus and pulmonary blood flow, which raises the volume burden on the left heart. Other possible complications of left-to-right shunting are steal syndrome, necrotizing enterocolitis (NEC), intraventricular hemorrhage (IVH), and death. Therefore, managing PDA is essential [3,4].

The echocardiogram (ECHO) is the gold standard for diagnosing and monitoring preterm neonates with PDA [5]. There are different treatment approaches in the management of PDA. The most commonly used treatment options are Nonsteroidal Anti-Inflammatory Medicines (NSAIDs), such as indomethacin or ibuprofen [6]. Surgical interventions might be considered if pharmacologic therapy fails. In the majority of current settings, transcatheter closure is favored over surgical ligation; nonetheless, long-term results are unsatisfactory, especially for preterm neonates [7].

Although ibuprofen and oral indomethacin are considered the first-line therapy for PDA, this approach is associated with various side effects, including renal damage, gastrointestinal issues, and platelet aggregation [8]. Current evidence suggests that a new strategy for closing PDAs involves using acetaminophen. Acetaminophen (also known as paracetamol in certain regions), a widely used analgesic and antipyretic, has shown promise as a non-invasive alternative to the traditional use of Ibuprofen. However, more high-quality studies are needed to compare the efficacy and safety of these two medications in this specific population.

Hammerman et al. published a case series on using acetaminophen to treat PDA in five neonates who had failed or were contraindicated from the ibuprofen course. Off-label oral acetaminophen (15 mg/kg every six hours) closed the ducts in all five neonates within 48 h [9]. The use of this approach in preterm neonates has increased substantially since then. Another attempt was made to assess the effect of intravenous (IV) acetaminophen on early ductus arteriosus closure and its potential adverse effects at a very early gestational age (32 weeks). Harkin et al. used a different approach, giving 20 mg/kg of acetaminophen after 24 h of life and 7.5 mg/kg every six hours for four days. The ductus closed 177 h after birth in treated individuals and 338 h in untreated individuals (*p* = 0.045). The authors state that acetaminophen promoted early closure of the ductus arteriosus without causing any adverse effects [10].

Several studies have examined the efficacy and safety of acetaminophen compared to ibuprofen. However, there are significant limitations. To date, the statistical confirmation of the effectiveness of the acetaminophen approach for this condition remains unresolved. Additionally, little consensus exists on using acetaminophen in preterm neonates who fail the initial course of ibuprofen.

Therefore, this study was conducted to test the hypothesis that acetaminophen is not inferior to ibuprofen for closing PDA shunts in preterm neonates. This study aimed to assess the efficacy and safety of the initial course of IV acetaminophen versus IV ibuprofen in closing PDA in preterm neonates. The result of this study could provide insights that optimize PDA management in preterm neonates.

## 2. Methods

### 2.1. Study Design and Setting

This retrospective cohort study was carried out at King Abdulaziz Medical City-Western Region, Ministry of National Guard Health Affairs. The Neonatal Intensive Care Unit (NICU) is a level III unit with 30 beds and utilizes a computerized physician order entry system across all inpatient wards. Ethical approval was obtained from the Institutional Review Board (Reg. No.: H-01-R-005).

### 2.2. Selection of Patients

All preterm neonates with a gestational age of 32 weeks or less who were admitted to the NICU between June 2016 and March 2021 and received IV acetaminophen 15 mg per kg every six hours for 3 to 7 days or IV ibuprofen 10 mg per kg followed by 5 mg per kg at 24 and 48 h, based on standard care for treating PDA, were included in the study. The hospital’s electronic database was used to screen for inclusion criteria. Neonates were excluded if they did not complete the treatment course, had other congenital heart anomalies, had ECHO reports missing, or died within 48 h of starting therapy.

### 2.3. Data Collection

The clinical characteristics, ECHO report, and orders for IV acetaminophen or IV ibuprofen were used to generate reports from the computerized database to identify neonates who met the inclusion criteria. After proper training, neonatal data were collected using a standardized data collection form. The information was de-identified and stored in a password-protected file. Additionally, the patients’ medical records were reviewed to confirm that the medications were administered to treat PDA. In our study, we classify PDA as large, moderate, or small using the El-Khuffash PDA Score. This score assesses ductal diameter, LA/Ao ratio, flow pattern, and systemic effects to evaluate hemodynamic significance. To assess the accuracy of the data, 10% of the entries were randomly checked. The data collected included: gestational age, postnatal age at the first dose, sex, weight, duct size before and after the intervention, length of NICU stay, type and dose of medication (acetaminophen or ibuprofen), duration of therapy, presence of renal failure or liver disease, baseline serum creatinine, liver enzymes (ALT, AST), platelet count, and any subsequent results obtained after starting treatment.

### 2.4. Outcomes

The primary goal of this study was to compare the effectiveness of first-course IV acetaminophen versus IV ibuprofen in closing PDA in preterm neonates. The secondary goals included assessing the effectiveness of the first vs. the second course of acetaminophen in closing the PDA, evaluating the safety profile of acetaminophen compared to ibuprofen, determining the rate of PDA re-opening, and identifying the need for surgical ligation. In our study, the first course was defined as the initial treatment with either ibuprofen or acetaminophen, while the second course was treatment with the same or a different agent if the initial attempt failed.

### 2.5. Statistical Analysis

The mean and standard deviation for normally distributed continuous data were used to describe the clinical characteristics of neonates in the acetaminophen and ibuprofen groups. For non-normally distributed continuous data, the median and interquartile ranges (IQR) were utilized. As categorical variables, frequency counts were used. The chi-squared test of independence (Χ^2^) was employed to determine whether the closed PDA success rates differed between ibuprofen and acetaminophen. A statistical *p*-value of ≤0.05 was considered significant. All statistical analyses were conducted using RStudio software (version 4.1.2).

## 3. Results

**Figure 1:**
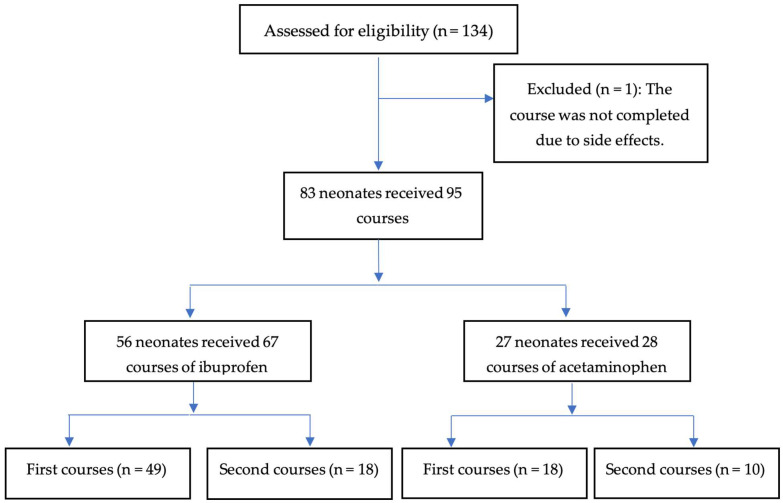
The flowchart of included patients.

The medical records of 134 neonates were reviewed for eligibility. Of these, 83 neonates met the inclusion criteria. According to the exclusion criteria, one neonate was excluded from the final analysis because they did not complete the course, and no patient died within 48 h of therapy. (Figure 1). Fifty-six neonates received 67 courses of Ibuprofen. In contrast, 27 neonates underwent 28 courses of acetaminophen.

Among the 83 neonates, there was no difference in gestational age and sex between the acetaminophen and ibuprofen groups. The baseline characteristics and findings of the preterm neonates are summarized in Table 1. All demographic parameters were comparable between the two groups, except for birth weight (951 g vs. 818 g), which had a *p*-value of 0.039. The first courses of PDA treatment were initiated at a mean age of 5.47 ± 10.30 days, and at 5.22 ± 6.43 days of life in the ibuprofen and acetaminophen groups, respectively. Most neonates in the IV ibuprofen group received a three-day treatment regimen (10 mg per kg followed by 5 mg per kg 24 and 48 h later). In the IV acetaminophen group, most neonates received a three or five-day treatment regimen (15 mg per kg every six hours).

In the group given acetaminophen, 5.5% had small PDA, 38.8% moderate, and 55.5% large. With ibuprofen, 4.1% had small PDA, 38.8% moderate, and 57.1% large. Consequently, no statistically significant differences were observed between the groups.

### Main Result

The rate of successful full PDA closure after the first courses was higher in the ibuprofen group than in the acetaminophen group. However, there was no significant difference between the two groups (71.4% vs. 55.6%, *p*-value = 0.35) as shown in Table 2. In 35 of the 49 neonates (71.4%), PDA was successfully closed after the first course of ibuprofen, and no further treatment was needed. After ibuprofen failed in one neonate, the ductus arteriosus was effectively closed with acetaminophen as a second therapy. The PDA was successfully closed in 10 of 18 neonates treated with the first course of acetaminophen and in 7 of 10 neonates treated with the second course of acetaminophen. Using acetaminophen as second-line therapy after ibuprofen failure resulted in PDA closure in 70%. While the initial use of acetaminophen closed 55% of PDAs, this difference was not statistically significant (*p*-value = 0.68). Regarding PDA severity, the proportions of neonates with small, moderate, and large PDA were similar between the acetaminophen and ibuprofen groups.

Surgical ductal ligation was performed in one neonate after two courses of ibuprofen. Another neonate who previously received acetaminophen followed by ibuprofen also required surgical ligation. The ductus arteriosus re-opening rates were similar between the ibuprofen and acetaminophen groups, with no significant difference (4% vs. 5.5%, *p* = 0.79). None of the neonates had to discontinue PDA treatment due to hepatic or renal damage. In Table 3, no significant difference was found between the groups before and after treatment regarding serum creatinine level, platelet count, UOP, ALT, and AST, as *p*-values were >0.05.

## 4. Discussion

Given the limited evidence supporting the use of acetaminophen as an alternative to ibuprofen for PDA closure, our study found that the rate of PDA closure with the first course of IV ibuprofen was higher than that with IV acetaminophen. However, the overall PDA closure rates were similar between both groups, with no statistically significant difference (*p*-value = 0.35). The success rate of PDA closure aligns with a recent randomized study by Meena et al., which reported that after the first and second courses of treatment, neonates receiving ibuprofen had a 77% success rate, compared to 71% for those receiving acetaminophen (*p* > 0.05) [11].

Our findings are similar to those of the following studies: A non-inferiority, randomized, multicenter study by Garca-Robles et al. examined the effectiveness and safety of acetaminophen and ibuprofen in 30-week-old premature neonates. The researchers concluded that acetaminophen is an effective alternative to ibuprofen [12]. Moreover, 100% PDA closure was reported by Oncel et al. after an IV acetaminophen course [13]. Furthermore, Oncel et al. found that the early course of treatment with oral ibuprofen and oral acetaminophen resulted in equal percentages of effective ductus closure (77.5% and 72.5%, respectively; *p* > 0.05) [14]. Similarly, El-Mashad et al. published a study comparing the effectiveness of acetaminophen and ibuprofen in preterm infants under 1500 g or 28 weeks of gestational age. The researchers found that acetaminophen closes PDA as effectively as ibuprofen, with an 80% closure rate compared to 77% for ibuprofen [1]. Huang et al. also conducted a meta-analysis of five randomized controlled trials on acetaminophen and ibuprofen for PDA in preterm neonates. The PDA closure rates were similar for both drugs (*p* = 0.62) [15]. Our findings also align with a systematic review by da Silva HDS et al., which examined 781 neonates with PDA treated with acetaminophen or ibuprofen. The review found that acetaminophen is equally effective and safe as ibuprofen for closing the DA, with no notable differences in effectiveness or side effects in most studies [16]. Because of this, acetaminophen may treat PDA as well as ibuprofen. In contrast to our findings, PDA closure was reported in only 18% of low-gestational-age neonates in a study conducted by Roofthooft et al., which found poor results with IV acetaminophen therapy as second-line treatment [17].

Compared to acetaminophen, ibuprofen therapy was linked to shorter PDA closure times. In our study, ibuprofen was initially administered at 10 mg/kg, followed by 5 mg/kg at 24 and 48 h. IV acetaminophen (15 mg per kg every six hours for three to seven days) was used for ductal closure treatment. However, there is significant variability in management. A duration of 3–7 days has been suggested for successful PDA closure [18]. While previous studies have demonstrated the effectiveness of oral agents, our study concentrated on IV therapy because of differences in patient characteristics. Most participants were extremely preterm, critically ill, and maintained nil per os (NPO) because of the high risk of gastrointestinal complications. Thus, IV therapy remains the standard care in our institution.

If the initial IV acetaminophen treatment is ineffective, management options include administering a second dose of acetaminophen or switching to IV ibuprofen, based on clinical judgment. In our study, administering acetaminophen as a second-line therapy after ibuprofen failure successfully closed the PDA by 70%. First course of acetaminophen treatment closed only 55% of PDAs, but this difference was not statistically significant (*p*-value = 0.68). This aspect of the study is important because there is little consensus on the appropriate timing for administering acetaminophen to preterm neonates who do not respond to initial ibuprofen therapy. One study indicated that acetaminophen is a safe and effective option for reducing the size and potentially treating hemodynamically significant PDA (hsPDA) after two weeks of age, when ibuprofen effectiveness diminishes [19]. Conversely, another study published in 2015 by Roofthooft et al. assessed the efficacy of acetaminophen for PDA closure in extremely low-birth-weight neonates and found it to be ineffective when given after failed ibuprofen treatment [14]. Additionally, El-Khuffash et al. conducted a retrospective review to evaluate the late effects of acetaminophen on PDA treatment prior to surgical ligation. Acetaminophen was administered at a median age of 27 days and successfully closed the PDA in only 9 of 25 neonates [20]. These findings highlight the importance of conducting future prospective studies to determine the most effective approach to PDA closure, either by repeating the same course or switching to a different agent, especially to evaluate how patient age and clinical characteristics influence treatment response.

Regarding safety, our study showed that acetaminophen and ibuprofen have similar safety profiles. It is well known that serious side effects of ibuprofen include decreased kidney function, gastrointestinal bleeding, and thrombocytopenia. Acetaminophen has also been associated with liver damage [21]. According to our findings, the rates of renal failure, liver disease, and thrombocytopenia did not differ significantly between groups. Similarly, other studies have concluded that both treatments are safe based on several markers of kidney and liver function, which aligns with our results [14,22]. A recent meta-analysis found that renal failure and gastrointestinal bleeding were more common in the ibuprofen group [23].

Ductus re-opening was observed in two cases following the first course of ibuprofen. However, there was no statistically significant difference in the re-opening rates between the two groups. Conversely, Oncel et al. reported that the oral acetaminophen group experienced a higher re-opening rate than the oral ibuprofen group. Nonetheless, this difference was not statistically significant (*p* = 0.43) [14].

In our cohort, the average gestational age at first treatment was about 27 weeks. PDA closure after the initial course occurred in 71.4% of neonates treated with ibuprofen and 55.6% with acetaminophen. Literature shows spontaneous closure rates of about 73% in neonates over 28 gestation weeks and 94% in infants over 1000 g at birth. More studies are needed to compare treated neonates with those who experienced natural closure, considering gestational age and weight [24,25]. This illustrates the variability in neonatal care practices and emphasizes the importance of tailored treatment approaches.

Our results align with a recent pilot randomized clinical trial published by Mukherjee et al. in 2026 involving preterm neonates with hsPDA who received IV acetaminophen and IV ibuprofen as rescue therapies for PDA closure. The study found that both drugs had similar effectiveness and safety profiles, with no significant differences in PDA closure rates or prematurity-related complications [26]. These results suggest that acetaminophen could be considered an effective and safe alternative to ibuprofen for the therapeutic closure of PDA in preterm neonates. This may reduce the need for more invasive procedures, such as surgical ligation, and the potential risks associated with them.

This study has certain limitations. It was conducted retrospectively, and the small sample size limited our ability to generalize the findings. In our study, there was variation in both gestational ages and chronological ages at the start of the treatment course when comparing ibuprofen to acetaminophen. Additionally, the study included different durations of therapy within the acetaminophen group. This heterogeneity could influence the efficacy and comparability of the treatment groups.

## 5. Conclusions

The study showed that the ibuprofen group was more likely to close the PDA than the acetaminophen group, although this difference was not statistically significant. The safety profiles of both treatments were similar. Given the nature of the study, a future randomized controlled trial is recommended to explore the clinical potential of acetaminophen for PDA closure and to examine the timing, optimal dosing, duration, and long-term outcomes of this therapy.

## Figures and Tables

**Table 1:** Baseline characteristics.

Characteristic	Ibuprofen (n = 67)	Acetaminophen (n = 28)	*p*-Value
Gestational age at first course, n (mean ± SD wk)	49 (27.4 ± 2.5)	18 (27.2 ± 2.5)	0.928
Neonatal age at each treatment course			
First course, n (mean ± SD days)	49 (5.47 ± 10.30)	18 (5.22 ± 6.43)	0.923
Second course, n (mean ± SD days)	18 (10.61 ± 6.39)	10 (15.70 ± 8.62)	0.086
Gender, n (%) Boy	34 (50.7)	15 (51.7)	0.456
Girl	33 (49.3)	14 (48.3)	
Birth weight, (g)	951.58 ± 334.99	818.15 ± 262.58	0.039
Length of NICU Stay, (d)	82.41 ± 45.8	74.0 ± 41.1	0.982
Duration of therapy (days), n (%) 3	63 (94.0)	9 (31.0)	--
4	0 (0)	5 (17.2)	
5	3 (4.5)	9 (31.0)	
6	1 (1.5)	2 (6.9)	
7	0 (0)	4 (13.8)	
Duct diameter before treatment, n (%)			
Small PDA	3 (4.48)	1 (3.5)	0.970
Moderate PDA	24 (35.8)	12 (42.8)	0.687
Large PDA	40 (59.7)	15 (51.7)	0.596

SD: Standard deviation.

**Table 2:** Treatment outcomes of PDA closure.

Outcomes	Ibuprofen (49)	Acetaminophen (18)	*p*-Value
First-course PDA closure, n (%)	49 (71.4%)	18 (55.6%)	0.35
Ductus re-opening, n (%)	2 (4%)	1 (5.5%)	0.79

**Table 3:** Laboratory values before and after the treatment in the two groups.

Treatment	Ibuprofen			Acetaminophen		
	Before Mean ± SD	After Mean ± SD	*p*-Value	Before Mean ± SD	After Mean ± SD	*p*-Value
Serum Creatinine (μmol/L)	60.13 ± 15.9	61.5 ± 13.9	0.859	64.13 ± 28.9	69.4 ± 33.4	0.141
Urine output (mL per Kg per hour)	3.36 ± 1.0	3.4 ± 0.8	0.518	3.19 ± 1.3	3.5 ± 1.1	0.258
Platelet Count (10^9^/L)	227.42 ± 118.4	222.3 ± 130.9	0.288	219.72 ± 135.2	207.0 ± 83.5	0.738
Alanine Transaminase (U/L)	8.6 ± 5.3	8.7 ± 5.5	0.33	22.73 ± 48.9	16.9 ± 27.2	0.277
Aspartate Transaminase (IU/L)	39.26 ± 20.6	32.9 ± 30.8	0.827	132.0 ± 434.5	55.5 ± 93.4	0.410

## Data Availability

The data presented in this study are available on request from the corresponding author. The data are not publicly available due to privacy and ethical reasons.
